# Persistence of Platelets Activation Prior to Second Doses of Covid-19 Vaccine After Vaccine-Induced Immune Thrombotic Thrombocytopenia

**DOI:** 10.1177/10760296221086283

**Published:** 2022-03-11

**Authors:** Guillaume Roberge, Marc Carrier

**Affiliations:** 1 Department of Medicine, 36896 Centre Hospitalier Universitaire de Québec, Université Laval, Hôpital Saint-François d’Assise, Québec, Canada; 2 Department of Medicine, 10055 Ottawa Hospital Research Institute, University of Ottawa, Ontario, Canada

A recent letter to the editor published in the New England Journal of Medicine reports the safety of administration of a second dose of Covid-19 vaccine in patients with vaccine-induced immune thrombotic thrombocytopenia (VITT) following ChAdOx1 nCoV-19. None of the 40 cases who received a second dose has developed complication regardless of the vaccine received.^1^ However, it is unclear if these patients were anticoagulated at that time or if platelet-activation assays remained positive prior to vaccination. Most of VITT cases shows normalization of platelet activation after 12 weeks.^2^ However, positive platelet-activation assay at 3 months of confirmed VITT with further related complications, suggesting higher thrombosis risk, have been previously described.^3^ One patient with positive platelet-activation assay before getting a second dose of Covid-19 vaccine while still receiving anticoagulation has also been reported, but no adverse events occurred.^2^ Although mRNA vaccines are not associated with VITT, the physiopathology is not fully understood and, hence, it is still unclear if a second dose of Covid-19 vaccine in patients with positive platelet-activation assay is safe.^4,5^ Furthermore, anticoagulation at that time could potentially mitigate the heightened risk of recurrent thrombosis. Clarification on these characteristics in the 40 described patients would help clinicians to discuss risks and benefits of a second dose of covid-19 vaccine for these patients.
